# Overview of the functional virulent genome of the coffee leaf rust pathogen *Hemileia vastatrix* with an emphasis on early stages of infection

**DOI:** 10.3389/fpls.2014.00088

**Published:** 2014-03-14

**Authors:** Pedro Talhinhas, Helena G. Azinheira, Bruno Vieira, Andreia Loureiro, Sílvia Tavares, Dora Batista, Emmanuelle Morin, Anne-Sophie Petitot, Octávio S. Paulo, Julie Poulain, Corinne Da Silva, Sébastien Duplessis, Maria do Céu Silva, Diana Fernandez

**Affiliations:** ^1^Centro de Investigação das Ferrugens do Cafeeiro/BioTrop/Instituto de Investigação Científica TropicalOeiras, Portugal; ^2^Computational Biology and Population Genomics Group, Centro de Biologia Ambiental, Faculdade de Ciências da Universidade de LisboaLisboa, Portugal; ^3^Institut National de la Recherche Agronomique, Centre INRA Nancy Lorraine, UMR 1136 INRA/Université de Lorraine Interactions Arbres/Micro-organismesChampenoux, France; ^4^Université de Lorraine, UMR 1136 INRA/Université de Lorraine Interactions Arbres/Micro-organismes, Faculté des Sciences et TechnologiesVandoeuvre-lès-Nancy, France; ^5^Institut de Recherche pour le Développement, UMR 186 IRD-Cirad-UM2 Résistance des Plantes aux BioagresseursMontpellier, France; ^6^Genoscope, Centre National de Séquençage, Commissariat à l'Energie Atomique, Institut de GénomiqueEvry, France

**Keywords:** appressorium, coffee leaf rust, germinating urediniospore, haustorium, pyrosequencing, transcriptome

## Abstract

*Hemileia vastatrix* is the causal agent of coffee leaf rust, the most important disease of coffee *Arabica*. In this work, a 454-pyrosequencing transcriptome analysis of *H. vastatrix* germinating urediniospores (gU) and appressoria (Ap) was performed and compared to previously published *in planta* haustoria-rich (H) data. A total of 9234 transcripts were identified and annotated. Ca. 50% of these transcripts showed no significant homology to international databases. Only 784 sequences were shared by the three conditions, and 75% were exclusive of either gU (2146), Ap (1479) or H (3270). Relative transcript abundance and RT-qPCR analyses for a selection of genes indicated a particularly active metabolism, translational activity and production of new structures in the appressoria and intense signaling, transport, secretory activity and cellular multiplication in the germinating urediniospores, suggesting the onset of a plant-fungus dialogue as early as at the germ tube stage. Gene expression related to the production of carbohydrate-active enzymes and accumulation of glycerol in germinating urediniospores and appressoria suggests that combined lytic and physical mechanisms are involved in appressoria-mediated penetration. Besides contributing to the characterization of molecular processes leading to appressoria-mediated infection by rust fungi, these results point toward the identification of new *H. vastatrix* candidate virulence factors, with 516 genes predicted to encode secreted proteins.

## Introduction

Rust diseases have been a long standing threat for centuries and have reshaped cultivation of crops and breeding strategies. Coffee leaf rust caused by *Hemileia vastatrix* Berk & Broome is the major disease of Arabica cultivated coffees (*Coffea arabica* L.) (Silva et al., 2006). *H. vastatrix* is considered as one of the most primitive phylogenetic lineages of the Pucciniales (Aime, 2006; Silva et al., 2012) and has no alternate host known so far. Since the 19th century, when it caused suppression of the coffee cultivation in Sri Lanka, the disease gained a worldwide distribution, reaching nearly all regions of the world where coffee is grown with severe economical damages. Breeding and selection of coffee resistant genotypes to different fungal races from several parts of the world has been successful (Silva et al., 2006), but as a consequence of the high adaptive potential of the pathogen, the emergence of new rust pathotypes and the corresponding breakdown of resistance has been observed in many improved coffee varieties in several countries (Várzea and Marques, 2005; Diniz et al., 2012; Cressey, 2013). Thus, currently coffee leaf rust still stands as the major constraint to Arabica coffee production.

During infection of their hosts, rust fungi differentiate several specialized infection structures such as germtubes, appressoria, stomatal vesicles, infection hyphae, haustoria, and spore-forming cells. Until recently, most of the biological knowledge gained at the molecular level on rust fungi was derived from EST sequencing, mainly from ungerminated and germinating urediniospores, rust-infected tissues, isolated haustoria and some spore types at other stages of their complex life cycle (for a review see Duplessis et al., 2012; Fernandez et al., 2013). However, some differentiation stages are not sufficiently covered yet and lack description and information, such as appressoria formation (Fernandez et al., 2013). For instance, two studies conducted in *Phakospora pachyrhizi* and *Puccinia triticina* reported that a high proportion of genes of unknown functions were expressed at the appressorial stage (Hu et al., 2007; Stone et al., 2012). Appressoria may be also differentiated by other pathogenic fungi, enabling host cuticle penetration through physical and/or chemical mechanisms. Key features of these specialized structures include the production of an extracellular matrix for adhesion to the surface, the accumulation of molar concentrations of glycerol for generating turgor pressure and the differentiation of a penetration hypha (Deising et al., 2000). Appressoria differentiated from urediniospores typically form over host stomata, and a penetration hypha is subsequently formed at the base of the appressorium to invade the substomatic chamber. There are evidences that mechanical pressure (about 0.35 MPa) is exerted by the penetration hypha when penetrating the stoma (Terhune et al., 1993). This is considerably less than the pressure exerted by some fungi that penetrate directly through the cuticle, such as *Magnaporthe oryzae* or *Colletotrichum* spp. (Howard et al., 1991; Chen et al., 2004), but enough to distort stomatal guard cell lips (Terhune et al., 1993). Nevertheless, rust fungi must also possess machinery for lytic penetration of host cuticle and cell wall, since hyphae produced by germinating basidiospores are capable of direct penetration into host epidermal cells (Voegele et al., 2009).

Until very recently, no genomic resources were available for *H. vastatrix*. After several years of lagging behind other rust fungi on genomic research, Fernandez et al. (2012) reported on the 454-transcriptome sequencing of rust-infected coffee leaves. This study generated 22,774 contigs of which 30% were assigned to *H. vastatrix*. Analysis of these *in planta* expressed sequence tags (ESTs) revealed that the majority (60%) had no homology in public genomic databases, representing potential coffee rust-specific genes. Nevertheless, *H. vastatrix* candidate effectors likely related to host infection and orthologous to other rust fungi, were identified among 382 predicted secreted proteins (Fernandez et al., 2012). Still, there is no knowledge of transcripts expressed at early stages of infection that could provide a more integrative scenario on the molecular mechanisms governing this pathosystem.

Complementing the knowledge gained into the *in planta* transcriptome of coffee rust, here we report on the in-depth transcriptome analysis of *H. vastatrix* by 454-based RNA-Seq during urediniospore germination and appressorium formation, two early and key stages of infection. Comparison of these specific stages with infected leaves allows an integrative characterization of transcript expression profiles during the course of biotrophic growth and infection. In particular, the identification of genes related with appressorium formation leads to novel insights into a stage that has been poorly described at the molecular level.

## Materials and methods

### Biological material, RNA isolation and cDNA synthesis

*Hemileia vastatrix* isolate CIFC 178a (race XIV: genotype *v*_2_ *v*_3_ *v*_4_ *v*_5_) was multiplied on its differential host plant (*C. arabica* accession CIFC H147/1, carrying the resistance factors *S*_*H*_2, *S*_*H*_3, *S*_*H*_4 and *S*_*H*_5). An *in vitro* method was used to produce germinating urediniospores and appressoria to ensure the generation of cDNA libraries with no contaminating plant sequences (Azinheira et al., 2001; Vieira et al., 2012). For the germinating urediniospores sample (gU), 19 mg of spores were spread in sterile distilled water in Petri dishes and incubated for 18 h at 24°C under darkness. For the appressoria sample (Ap), 15 mg of spores were spread over oil-collodion membranes (Vieira et al., 2012) in Petri dishes, sprayed with water and incubated for 24 h at 24°C and 100% relative humidity, under darkness. For an accurate sample characterization, urediniospore germination and appressoria formation were quantified, showing that the germinating urediniospores sample (gU) comprised over 50% of germinating urediniospores. The appressoria sample (Ap) comprised over 60% of germinating urediniospores with appressoria. These are considered rather fair rates for *H. vastatrix* (Azinheira et al., 2001).

Samples gU and Ap were harvested, immediately frozen in liquid nitrogen and the RNA was isolated from each sample with the RNeasy Plant minikit (Qiagen, Hilden, Germany), including an in-solution DNase treatment following the manufacturer's instructions. RNA concentration and integrity were evaluated by spectrometry (Lambda EZ201, Perkin-Elmer, Waltham-MA, USA) and capillary electrophoresis (Bioanalyzer 2100, Agilent, Santa Clara-CA, USA) respectively. Following the SMARTer Pico PCR cDNA Synthesis Kit (Clontech, Saint-Germain-en-Laye, France) protocol, cDNA were synthesized from 1 μg total RNA using SMARTScribe Reverse Transcriptase (Clontech) and amplified using Advantage 2 Polymerase (Clontech). cDNA fragments, which ranged between 500 and 3000 bp, were purified with the NucleoSpin Extract II kit (Macherey-Nagel, Düren, Germany) and their quality and concentration were evaluated by electrophoresis.

### Pyrosequencing and assembly of 454 reads

For each sample, 20 μg cDNA was used for 454-pyrosequencing run on half of a picotitre plate on a Genome Sequencer FLX System using long-read GS FLX Titanium chemistry (Roche; www.454.com) at the Genoscope (Centre National de Séquençage, Evry, France; www.genoscope.cns.fr) following standard procedures recommended by Roche.

Raw sequences obtained for gU and Ap samples were assembled into contigs using Newbler 2.5 (Roche) with default parameters. For comparative purposes, the MIRA 3.2 assembler (http://sourceforge.net/apps/mediawiki/mira-assembler) was also employed. The relative abundance (Ra) of transcripts was calculated as the ratio between the number of 454 reads per contig and the length of the assembled contig (Vega-Arreguín et al., 2009).

### Bioinformatic analysis of transcripts

As previously described (Fernandez et al., 2012), sequence homology searches were performed against several databases: the NCBI non-redundant (nr) nucleotide and protein databases (www.ncbi.nlm.nih.gov), the genome sequences of *Melampsora larici-populina* and *Puccinia* spp. (Cantu et al., 2011; Duplessis et al., 2011a; www.jgi.doe.gov and www.broadinstitute.org, respectively); the euKaryotic Orthologous Group (KOG) database (Tatusov, 2003); the Pathogen-Host Interaction (PHI-base v3.2) reference database (Winnenburg et al., 2007; www.phi-base.org); the Phytopathogenic Fungi and Oomycete EST Database (COGEME v1.6; Soanes and Talbot, 2006); and a Pucciniales EST database (168,199 ESTs retrieved from GenBank in November 2012—unchanged number as of December 2013). Besides these, 16,831 transcripts from the *M. larici-populina* frozen gene catalog (http://genome.jgi-psf.org/Mellp1/Mellp1.download.ftp.html) and 20,567 (*P. graminis* f. sp. *tritici*) and 11,638 (*P. triticina*) from the *Puccinia* spp. transcript catalogue (http://www.broadinstitute.org/annotation/genome/puccinia_group/MultiDownloads.html) were also considered. Homology searches were performed using BLAST algorithms (Altschul et al., 1997) with a cut-off criterion (*e*-value < 10^−5^). For each search against a given database, only the best hit was considered. The assignment of 454-contig sequences into KOG functional categories was obtained using Reverse psi-BLAST (RPSBLAST; Altschul et al., 1997) against the KOG database.

Open reading frames (ORFs) were predicted with the translation tool getorf from the European Molecular Biology Open Software Suite (EMBOSS; http://emboss.bioinformatics.nl/cgi-bin/emboss/getorf) using default parameters. ORFs below 18 amino acids were not considered. A secretome bioinformatics pipeline was employed to define a tentative set of secreted proteins encoded by *H. vastatrix* transcripts, using SignalP v4.0 (Petersen et al., 2011), TargetP v1.1 (Emanuelsson et al., 2000) and TMHMM v2.0 (Krogh et al., 2001).

The catalytic and carbohydrate-binding modules (or functional domains) of enzymes that degrade, modify, or create glycosidic bonds (carbohydrate-active enzymes—CAZymes) was investigated by blastp comparison of predicted polypeptides to the CAZymes database (www.cazy.org; Cantarel et al., 2009) and to the CAZymes from *M. larici-populina* and *P. graminis* f. sp. *tritici* (Duplessis et al., 2011a). Similarly, proteins involved in membrane transport were investigated by blastp searches against predicted polypeptides in the Transporter Classification Database (www.tcdb.org; Saier et al., 2006, 2009).

Contigs from gU and Ap samples, as well as those predicted as fungal from a 21 day infected-coffee leaf sample (sample H; Fernandez et al., 2012), were compared using a best reciprocal BLAST hit approach with BioEdit 7.0.4.1 (Hall, 1999). Pairs of contigs with an *e*-value lower than 10^−30^ were considered as representing the same transcript and assembled. Ra values were calculated for each transcript present in more than one library, and these values were compared across the libraries in order to evaluate variations in expression levels. For such, τ values were calculated for each gene based on the normalized Ra values, in order to account for differences in library sizes. The expression specificity index (τ) is defined as τ = $\frac{\sum_{i\;=\;1}^{n}\left(1\;-\;x_{i}\right)}{n\;-\;1}$, where n is the number of tissues and x_*i*_ is the expression profile component normalized by the maximal component value (Yanai et al., 2005). The genes with the most stable expression across the three libraries were selected (105 genes with τ values below 0.25). Average Ra values were calculated among these genes for each library (0.02684 for gU, 0.03158 for Ap and 0.03039 for H) and Ra values for each contig in each library were normalized to the gU sample, following the strategy described by Ekblom et al. (2010) based on the guidelines provided by Mank et al. (2008).

### RT-qPCR

Germinating urediniospores (gU) and appressoria (Ap) samples for *H. vastatrix* isolate 178a were obtained as described above. *In planta* time course samples were collected at 18 h (mostly containing appressoria) and 1, 2, 3, 7, 14, and 21 days after inoculation for the compatible interaction between isolate 178a and the *C. arabica* genotype H147/1, as previously described (Diniz et al., 2012; Vieira et al., 2012). Fungal germination, appressoria formation and the differentiation of infection structures *in planta* were monitored by light microscopy as previously described (Vieira et al., 2012). RNA extraction, cDNA synthesis and RT-qPCR experiments were performed as previously described (Vieira et al., 2012), using Hv00099, 40 S ribosomal protein and glyceraldehyde-3-phosphate dehydrogenase as reference genes (Vieira et al., 2011) and ungerminated urediniospores as the control sample. A set of 43 genes was selected for RT-qPCR analysis based on RNA-Seq expression profiles and assigned functions. Primers (Supplementary Data 1) were designed as previously described (Vieira et al., 2012).

## Results and discussion

### 454-Pyrosequencing data for germinating urediniospores and appressoria samples

Given that different 454-pyrosequencing data assemblers are available and are known to generate diverse results (Kumar and Blaxter, 2010), MIRA 3.2 and Newbler 2.5 were compared in this study. In general, MIRA produced shorter and more numerous contigs. The overall homology scores of contigs to a Pucciniales EST database was better for the Newbler assembly (data not shown), suggesting a better quality of the assemblage which led us to use Newbler assembly in this study.

For samples gU and Ap (Table 1), a total of 455,807 sequence reads (113,404,366 nucleotides) was generated and assembled into 9108 contigs (4267 for gU and 3626 for Ap), with ca. 24% sequences remaining as either too short/low quality sequences (7%) or singletons (17%). Among those, 1214 contigs (13%) were <100 bp and not further considered in the analysis. The remaining 7894 contigs (Supplementary Data 2) had a mean length of 656 bp (Table 1), with 16% contigs larger than 1 kb (3.7% larger than 2 kb). Mean number of reads per contig was 41.0, with 11% contigs over 50 reads. Mean relative abundance (Ra) was 0.1153, with 18% contigs (1424) representing transcripts with a medium to high rate of expression (Ra > 0.05).

**Table 1:** **Descriptive statistics for *Hemileia vastatrix* 454 pyrosequenced cDNA libraries of germinated urediniospores (gU) and appressoria (Ap)**.

**Library**	**gU**	**Ap**
Number of bases	67773266	45631100
Number of sequences	269199	186608
Mean size of reads (bp)	251.8	244.5
Number of contigs	4267	3626
Mean size of contigs (bp)	676	632
Size of contigs (bp)^*^	188/546/1293/3754	192/530/1139/4860
Mean number of reads per contig	48.5	32.2
Reads per contig^*^	4/10/63/3326	4/9/52/2077
Mean relative abundance (Ra)	0.1456	0.0797
Relative abundance (Ra)^*^	0.0087/0.0190/0.0967/26.72	0.0085/0.0187/0.0777/14.41

* *Values correspond to 10/50/90/100 percentiles*.

In the absence of genomic information for *H. vastatrix*, contigs were compared to sequences deposited in databases (summary in Table 2 and results by contig listed in Supplementary Data 2), and 54% contigs had homology (*e*-value < 10^−5^) to the NCBI nr nucleotide database using blastn (Supplementary Data 2, columns G–I).

**Table 2:** ***Hemileia vastatrix* transcript homology in databases and KOG functional categories classification**.

**Library**	**gU**	**Ap**	**Total**	**% of all gU contigs**	**% of all Ap contigs**	**% of all contigs**
NCBI nr_blastn	2119	2126	4245	49.66	58.62	53.78
Mlp genome_tblastx	2356	2159	4515	55.21	59.53	57.20
Pgt_genome_tblastx	2294	2099	4393	53.76	57.87	55.65
Pt_genome_tblastx	2410	2171	4581	56.48	59.86	58.03
Pst_genome_tblastx	2408	2159	4567	56.43	59.53	57.85
EST_Pucciniales_tblastx	2507	2334	4841	58.75	64.35	61.33
SwissProt_blastx	1301	1362	2663	30.49	37.55	33.73
PHIbase_tblastx	482	444	926	11.30	12.24	11.73
COGEME_tblastx	2016	2042	4058	47.25	56.30	51.41
KOG	1691	1690	3381	39.63	46.59	42.83
Posttranslational modification, protein turnover, chaperones				12.5	13.6	
Translation, ribosomal structure and biogenesis				12.4	19.7	
Intracellular trafficking, secretion, and vesicular transport				8.2	5.7	
Energy production and conversion				7.9	9.1	
Signal transduction mechanisms				7.5	6.3	
Lipid transport and metabolism				5.7	6.4	

*Summary of the number and percentage of hits in homology searches of the H. vastatrix germinated urediniospores (gU) and appressoria (Ap) contig libraries against the NCBI nr database (NCBI nr_blastn), rust genomic and transcriptomic databases (Mlp_genome_tblastx, Pgt_genome_tblastx, Pt_genome_tblastx, Pst_genome_tblastx, EST_Pucciniales_tblastx), the SwissProt database (SwissProt_blastx) and functional databases (PHIbase_tblastx, COGEME_tblastx, KOG)*.

A total of 13,951 sequences obtained from the 21-days *H. vastatrix* infected-coffee leaf samples (H library) and previously predicted as of plant origin (Fernandez et al., 2012) were compared to the gU+Ap sequences, from which 22 showed an homology *e*-value below 10^−60^ (19 of which had *e*-value of 0.0; Supplementary Data 3). This analysis showed that only 0.1% of the sequences predicted as of plant origin (Fernandez et al., 2012) were wrongly assigned to this class. Similarly, among 2060 contigs previously classified as “not attributed/not resolved,” 28 had homology to gU+Ap sequences with an *e*-value below 10^−60^ (21 of which had *e*-value of 0.0). These 50 contigs (*e*-value <10^−60^) were incorporated in the present study, together with the 4415 fungal contigs initially identified in the H library, summing a total of 4465 H contigs (Fernandez et al., 2012) to our dataset.

### Comparison to the *in planta* expressed fungal sequences

**Figure 1:**
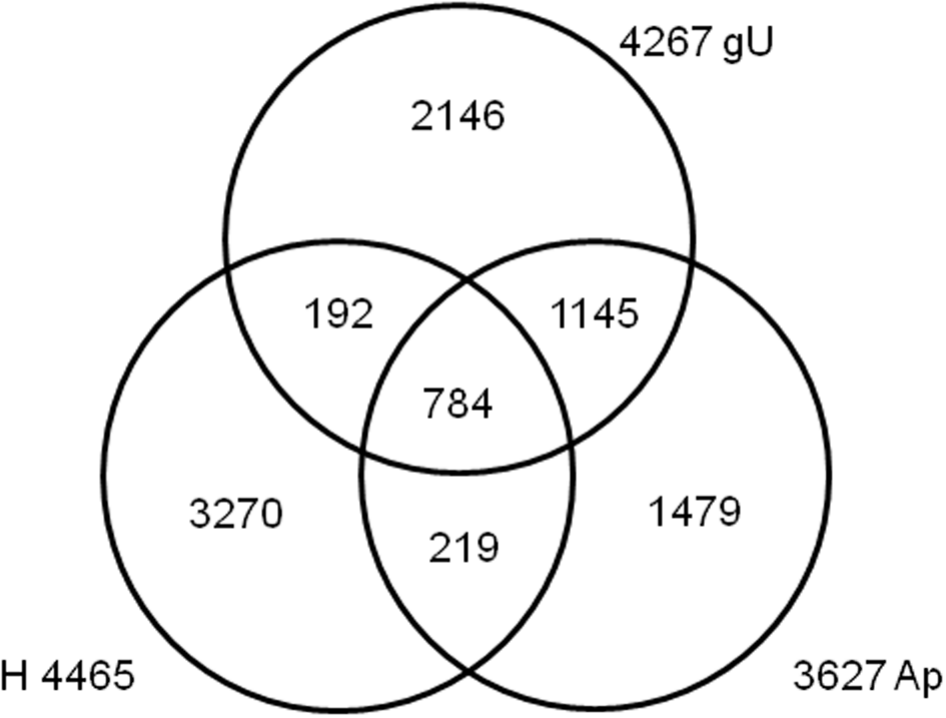
**Venn diagram depicting the comparison of *Hemileia vastatrix* EST libraries, using a best reciprocal BLAST hit strategy**. Number of contigs is indicated for the germinating urediniospores (gU), appressoria (Ap) and infected leaves 21 days after inoculation (H) libraries.

A best reciprocal BLAST strategy was used to compare the contigs from both gU and Ap libraries, as well as the fungal contigs from the H library (Fernandez et al., 2012). This enabled the identification and re-assembly of 784 sequences shared by the three libraries, 1145 shared only by gU and Ap, 219 by Ap and H and 192 by gU and H (Figure 1 and Supplementary Data 4, columns B–D). The remaining 6894 sequences (75%) are exclusive of each library. Altogether, 9234 unique *H. vastatrix* sequences were identified, which represents >50% of the total number of genes predicted from the genomes of *M. larici-populina* (16,399 genes) and *P. graminis* f. sp. *tritici* (17,773 genes) (Duplessis et al., 2011a). In order to further ascertain a measure of the genome coverage obtained in this study, we compared each of the three libraries (gU, Ap, and H) separately, along with the total set of 9234 sequences, against the FUNYBASE database containing 246 families of single-copy orthologs obtained from 21 genomes (core fungal genes) (Marthey et al., 2008). Each individual library contained only about half of the 246 genes (39% in H and 55% both in Ap and gU; data not shown), but the gU+Ap+H library included 174 (71%) of those core genes (Supplementary Data 4, columns BN-BP). These results indicate that the 9234 unique *H. vastatrix* transcripts provide a significant coverage of the *H. vastatrix* functional genome.

According to their RNA-Seq expression values and assigned functions, the expression profiles of a set of 43 genes was analyzed by RT-qPCR along the time course of a compatible interaction (Table 3).

**Table 3:**
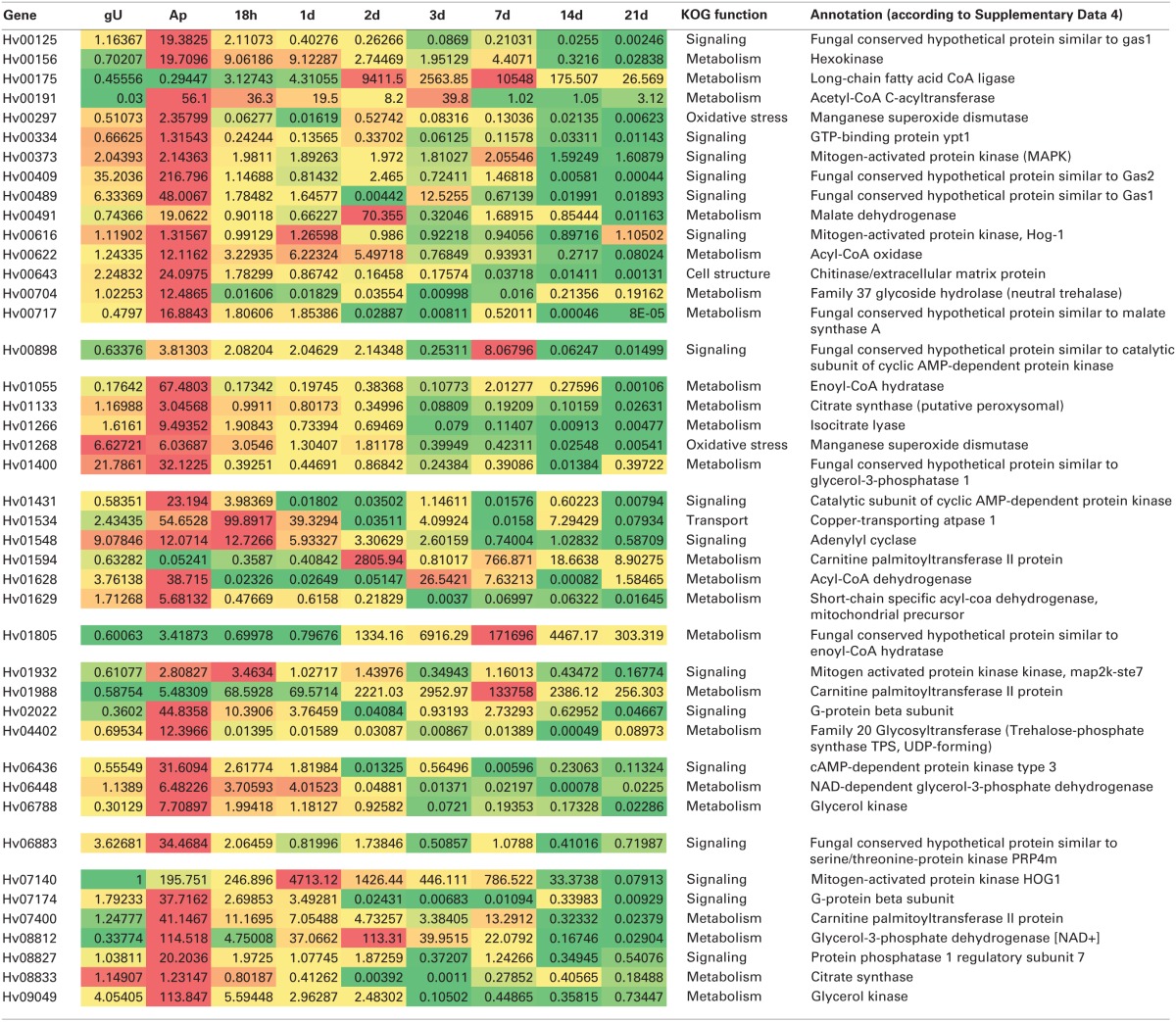
**Heatmap of *Hemileia vastatrix* genes expression profiles in germinating urediniospores (gU), *in vitro*-obtained appressoria (Ap) and *in planta* samples (from 18h to 21d after inoculation) obtained by RT-qPCR by comparison with 454 pyrosequencing-derived relative abundance transcript levels**.

*Values represent “fold change” related to the levels of expression in resting urediniospores (except for transcript 07140, where germinating urediniospores were used because of no amplification was obtained in resting urediniospores); Color scale: green to red denote lowest to highest expression levels across time points for each gene*.

### Gene function

Over 72% of the 9234 *H. vastatrix* transcripts had no specific KOG category assigned (No hits, “Function unknown” or “General function prediction only”; Table 4 and Supplementary Data 4, columns BK-BM). Within the remaining transcripts, the most represented KOG categories are “Translation, ribosomal structure and biogenesis” (14%) and “Post-translational modification, protein turnover, chaperones” (12%), while other nine categories represent 5–8% each.

**Table 4:**
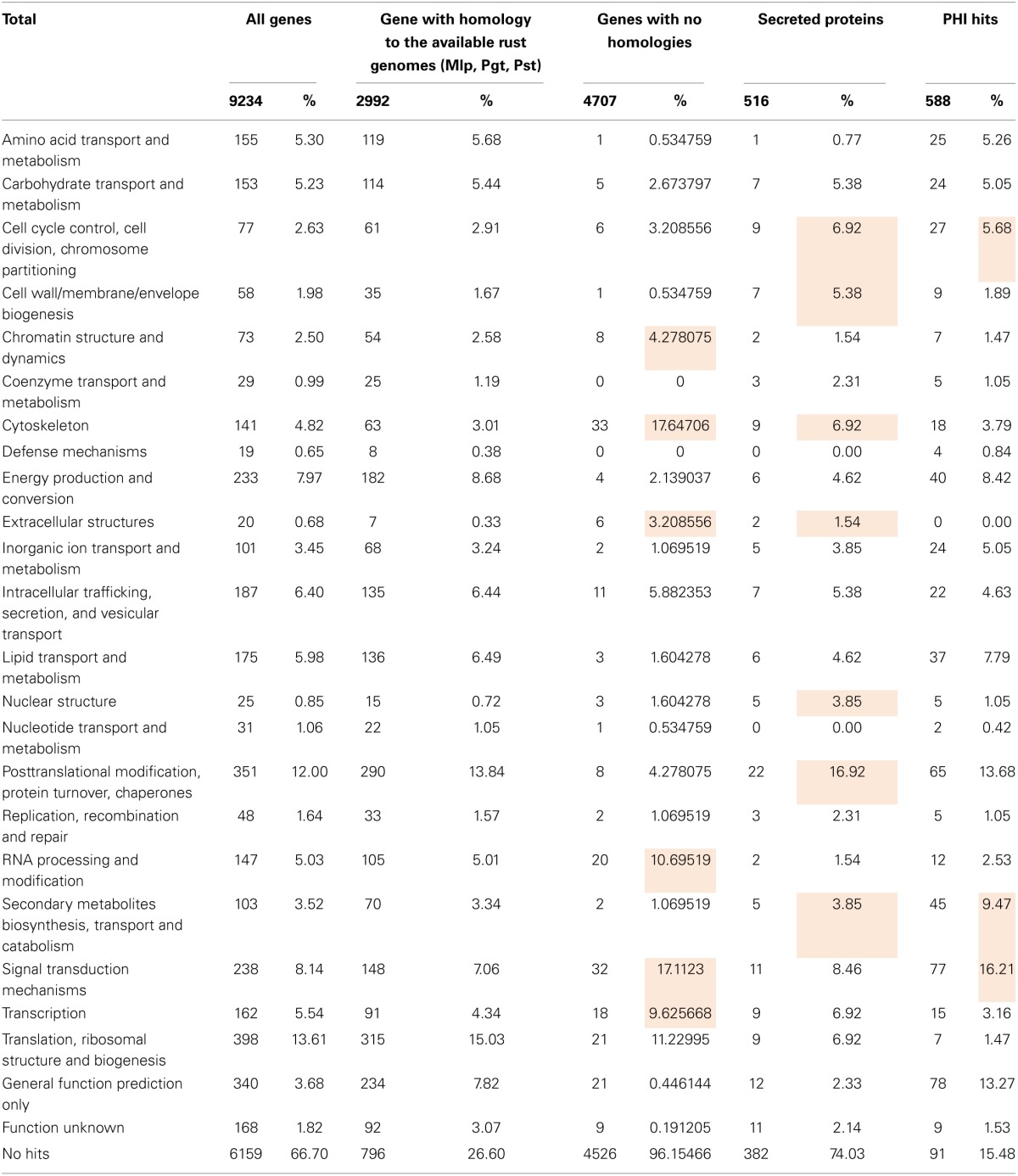
**Distribution of *Hemileia vastatrix* genes by KOG categories considering all genes and different groups of genes according to their homology or predicted function**.

*For the non-specific categories (“No hits,” “Function unknown” or “General function prediction only”), % refers to the total number of genes. For the remaining categories, % refers to the total number of genes with specific KOG categories assigned; highlighted cells correspond to information that is referred to in the article*.

A total of 4040 transcripts (44% of total) presented homologies against the NCBI non-redundant nucleotide database (Supplementary Data 4, columns V–X). A similar value (45%) was obtained by comparison (tblastx) with the Pucciniales EST database (Supplementary Data 4, columns AY-BA), with the most frequent organisms being *M. larici-populina*, *P. triticina*, *P. graminis* f. sp. *tritici* and *P. pachyrhizi* in similar proportions to those reported for the gU and Ap libraries (Supplementary Data 4, columns Y–AN). A total of 2992 transcripts (32%) have homology to all available rust genome sequences, suggesting that the corresponding genes are conserved among the Pucciniales. Only 16 transcripts showed homology to *P. graminis* f. sp. *tritici* or *P. triticina* mitochondrial sequences (Supplementary Data 4, columns AS-AX). A total of 141 and 148 transcripts did not show homology to *M. larici-populina* and *P. striiformis* gene models respectively, although showing significant homology to their genome sequences, which could indicate actual genes that were not predicted by automatic annotations in the corresponding genomes (Supplementary Data 4, columns AB–AE and AO–AR). Interestingly, 4707 transcripts (51% of total) showed no homology to the rust genes identified in genome sequences or EST databases (at a cut off *e*-value of 10^−10^), suggesting they may correspond to highly divergent or specific *H. vastatrix* genes. In fact, among these, only 3.2% have a specific KOG category assigned, with an overrepresentation of categories involved in cellular structure, nucleic acid activity and signaling (“Cytoskeleton,” “RNA processing and modification,” “Transcription” and “Signal transduction mechanisms”).

A total of 3573 transcripts showed homology to annotated fungal genes listed in the COGEME database (Supplementary Data 4, columns BH-BJ), *Ustilago maydis* (21%), *Giberella* spp. (13%), and *M. oryzae* (13%) being the most represented species. Further, 588 transcripts showed homology to fungal pathogenicity-related genes listed in the PHI database (Supplementary Data 4, columns BE-BG; 94% of which also have homologues in the COGEME database), mostly from *M. oryzae* (19%), *Candida albicans* (18%), and *Cryptococcus neoformans* (14%). About 70% of these 588 transcripts had specific KOG categories assigned, with categories such as “Cell cycle control, cell division, chromosome partitioning,” “Secondary metabolites biosynthesis, transport and catabolism” and “Signal transduction mechanisms” overrepresented as compared to KOG categories assigned to all genes (Table 4).

For each transcript, the size of the predicted polypeptide was compared to the size of the corresponding polypeptide in the *P. graminis* f. sp. *tritici* genome. Among the 3686 hits to the *P. graminis* f. sp. *tritici* genome, 24% were >90% the length of their orthologs (46% proteins were >50% size; Supplementary Data 4, columns BQ-BS).

As *H. vastatrix* 454 cDNA libraries were not normalized, the number of reads contained in each contig can be considered a relative expression level of each gene. For each contig, the number of reads was divided by the length of the contig, resulting in a Relative Abundance (Ra) index (Supplementary Data 4, columns G-O). Comparison among contigs from different libraries required a normalization step in order to account for differences in library sizes (Supplementary Data 4, columns P–S). Based on the comparative analysis of the expression levels identified in each library, nine different expression profiles were defined (Supplementary Data 4, column U). The most frequent profiles observed reflect the fact that 75% of contigs are exclusive of a single library, with 87% of the 9234 predicted transcripts presenting profiles 1, 2, or 3 (Table 5, row 4).

**Table 5:**
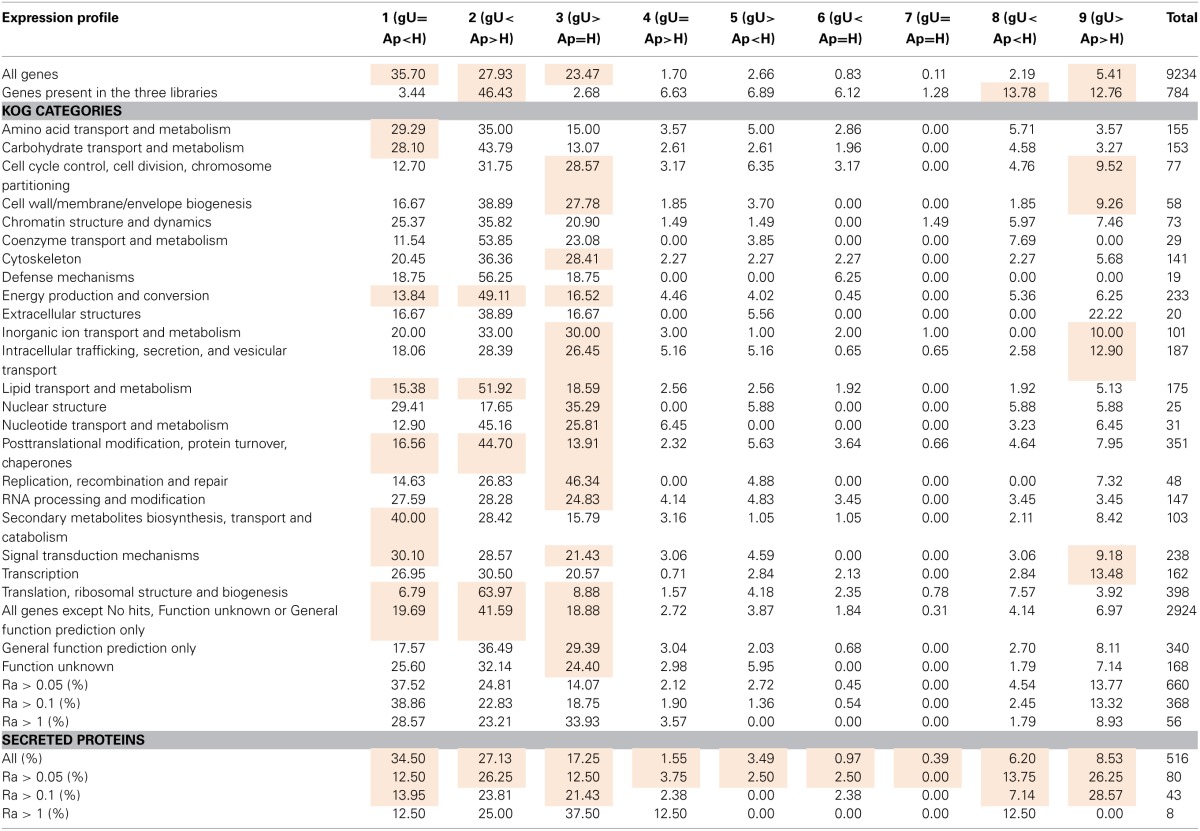
**Distribution (%) of *Hemileia vastatrix* genes by expression profile across the three differentiation stages, considering all genes and different groups of genes according to their predicted function and relative abundance**.

*Highlighted cells correspond to information that is referred to in the article*.

The analysis of relative abundance values according to the KOG category of each gene (Table 5) suggests a particularly active metabolism, translational activity and production of new structures in the Ap sample and both intense signaling and secretory activity and cellular multiplication in germinating urediniospores. In the H sample, over-represented KOG categories suggest intense signaling and nutrient uptake from the host to the fungus, as previously pointed out (Fernandez et al., 2012).

### Secreted proteins

**Figure 2:**
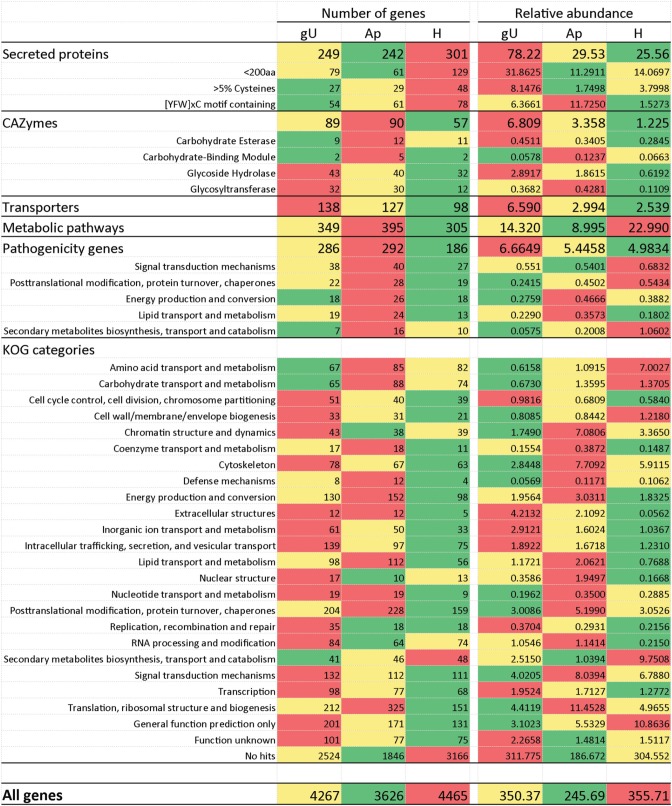
**Heatmaps of the number of genes and sum of their relative abundance values (= number of transcripts/transcript length) in the three libraries (germinating urediniospores, gU; appressoria, Ap; infected leaves 21 days after inoculation, H) for transcripts according to the main categories under analysis**. Color scale: green to red denote lowest to highest expression values for each gene.

A total of 467 putative secreted proteins were predicted with a secretion prediction pipeline composed of the SignalP, TargetP and TMHMM programmes (Supplementary Data 4, columns BT to CI for SignalP, CJ to CO for TargetP, CP to CS for TMHMM, CT for TMHMM vs. SignalP comparison and CU for final secretion prediction score). Besides these, other transcripts showing high homology (*e*-value < 10^−30^) to the *M. larici-populina* or the *P. graminis* f. sp. *tritici* predicted secreted proteins (Duplessis et al., 2011a) were also selected (Supplementary Data 4, column CV) even if not detected by the pipeline. Since these sequences were shorter than their orthologs, the signal peptide may be lacking from sequence. From this list of 516 transcript encoding putative secreted proteins, 87 and 70% entries presented less than 300 amino acids, and 200 amino acids, respectively (Supplementary Data 4, column BR). Also, 82 of these translated gene sequences are highly enriched in cysteine residues (5–15% of all amino acids; Supplementary Data 4, column CW), the vast majority of which (78) is less than 200 aa, similar to what was reported for *M. larici-populina* small secreted proteins (Hacquard et al., 2012). Nearly 60% of these 82 sequences contain a [YFW] × C motif (Supplementary Data 4, column CX), while only 20% of the remaining 434 sequences (<5% cysteines) possess that motif. An overrepresentation of this particular motif was similarly observed for the small secreted proteins of the poplar rust fungus (Hacquard et al., 2012). Secreted proteins transcripts tend to present high relative expression values: while they represent 5.6% of all 9234 genes in this study, they represent 12–14% of genes with Ra > 1 (Table 5). Moreover, the sum of all Ra values for predicted secreted proteins is higher in gU than in Ap or H, although more genes were identified in H (Figure 2). In addition, 46 of these genes encoding predicted secreted proteins present homology to genes in the PHI database (Supplementary Data 5) whose mutants in various fungi exhibit either loss of pathogenicity or reduced virulence phenotype, 21 of which were up-regulated in the gU library, 13 in Ap and 11 in H according to Ra values.

Four transcripts (00303, 00357, 01043, and 04304) encoding predicted secreted proteins are orthologous of the rust transferred protein (RTP1) genes (Pretsch et al., 2013) from *U. fabae*, *M. occidentalis* or *M. medusae* f. sp. *deltoidis* (Supplementary Data 6). Orthologs of these four genes were also identified in *M. larici-populina* and *Puccinia* spp. genomes (Supplementary Data 7) and the overall similarity among genes is quite low (Supplementary Data 6, columns Z–AD). Three of these transcripts were previously identified in *H. vastatrix* transcripts (Fernandez et al., 2012; see Supplementary Data 6). Transcript 04304 was exclusively detected in the H library (Supplementary Data 6), corroborating the observations by Vieira et al. (2012) and the expression profile of RTP1 in *U. fabae* (Kemen et al., 2005). Transcript 01043 was detected in Ap and H, and transcripts 00303 and 00357 were identified in the three libraries. Different expression profiles could be observed, transcript 00303 being highly expressed in Ap, transcript 01043 more expressed in Ap and H and transcript 00357 showing similar Ra values across the three libraries. *H. vastatrix* RTP1 orthologs show distinct expression profiles for the different members of this single gene family. Such an observation confirms the very dynamic and specific transcriptional process at the gene family level that was reported for gene families encoding small secreted proteins of *M. larici-populina* during time course infection of poplar leaves (Duplessis et al., 2011b).

Some *H. vastatrix* transcripts are orthologous of haustorially expressed secreted proteins (HESP) identified in *Melampsora lini* (Dodds et al., 2006; Barrett et al., 2009) (Supplementary Data 7). Among these, HESP-178 is orthologous to the transcripts 01506 and 04456, detected respectively in gU and Ap, and in H libraries (Supplementary Data 8). HESP-379 is orthologous to transcript 00258, which was identified in the three libraries at decreasing levels of expression along the differentiation stages, confirming previous observations (Fernandez et al., 2012). HESP-767 is orthologous to transcript 09298 only identified in library Ap. No homology was detected to *Melampsora* spp. Avr genes (Dodds et al., 2004), presumably because of their poor conservation across the Pucciniales (Catanzariti et al., 2006; Duplessis et al., 2011a).

Several transcripts present homology to genes involved in the alleviation of oxidative stress caused by ROS. By instance, two transcripts (00297 and 01268) with homology to Mn-type superoxide dismutase, orthologs of the *Cryptococcus gattii* pathogenicity-required manganese superoxide dismutase gene (*sod2*) (Narasipura et al., 2005) were identified. Transcript 00297 is ortholog of *U. fabae* gene *Uf058*, *P. graminis* f. sp. *tritici* gene PGTG_04728 and *M. larici-populina* gene 107563. These *H. vastatrix* genes are up-regulated in germinating urediniospores and their proteins are predicted to be secreted, suggesting an early role in response to plant defense responses (Table 3).

### CAZymes

The comparison of *H. vastatrix* transcripts to the carbohydrate-active enzymes (CAZymes) database (www.cazy.org; Cantarel et al., 2009) and to the predicted *M. larici-populina* and *P. graminis* f. sp. *tritici* CAZymes (Duplessis et al., 2011a) enabled the identification of 148 putative CAZymes in the coffee rust fungus. This number represents ca. 45 % of the CAZymes in the poplar and the wheat stem rust fungi genomes (Supplementary Data 9 and Supplementary Data 4, column DB), similar to those arising from the comparison of the total number of transcripts predicted in this study to the number of genes in those two genomes. However, the number of *H. vastatrix* CAZymes transcripts varies according to the type and family of enzymes. For instance, 13 and 14 genes belonging to the Glycoside Hydrolase family 47 were identified in *M. larici-populina* and in *P. graminis* f. sp. *tritici* respectively, while only two transcripts were detected in *H. vastatrix*. Several other gene families are found in comparable numbers in the three fungal species, even so in the most abundant families (e.g., Carbohydrate Esterase family 4, Glycoside Hydrolase families 5 and 16, Glycosyltransferase family 2). On the contrary, some transcript families found in *H. vastatrix* are absent from the genomes of *M. larici-populina* (e.g., Glycosyltransferase families 25 and 43) or *P. graminis* f. sp. *tritici* (Glycoside Hydrolase families 51 and 92). Additionally, eight Glycoside Hydrolase family 7 genes were identified both in *M. larici-populina* and in *P. graminis* f. sp. *tritici*, but none in *H. vastatrix* transcripts. CAZyme transcripts were more frequently expressed in the gU library and less in H (Figure 2). Among CAZymes transcripts identified in this study, 31 presented homology to genes in the PHI database.

### Transporters

A comparison to the Transporter Classification Database (www.tcdb.org; Saier et al., 2006, 2009) and to transporters from *M. larici-populina* and *P. graminis* f. sp. *tritici* (Duplessis et al., 2011a) enabled the identification of 215 transcripts encoding putative transporters. This represents ca. 60% of the number of transporters inferred from the *M. larici-populina* and *P. graminis* f. sp. *tritici* genome sequences (Duplessis et al., 2011a), again a similar proportion to that reported for other *H. vastatrix* transcript categories. However, deviations to this proportion occur in different transporter families. By instance, a family expansion is apparent in *H. vastatrix* for the F-ATPase family (H+- or Na+-translocating F-type, V-type and A-type ATPase) with 25 different transcripts predicted in *H. vastatrix*, against 20 in *M. larici-populina*, 22 in *P. graminis* f. sp. *tritici*, and 19–25 in a selection of basidiomycetes (Duplessis et al., 2011a). Similarly, variations in the Ra values can be related to the transporter type (Supplementary Data 10 and 11). In general, these results suggest that the transport capacity is at least as high in gU or Ap as in H.

Among the 215 *H. vastatrix* transcripts encoding putative transporters, 60 show homology to the PHI database. Both Ra and gene number are higher in gU and lower in H (Figure 2). Fourty are ATP-dependent transporters, including members of the ATP-binding cassette superfamily, (transcripts 01804, 07317, and 09267) and members of the P-type ATPase superfamily (transcripts 00176, 00302, 00402, 01534, and 07365), which are mostly expressed in the gU and Ap libraries. These results are corroborated by RT-qPCR for transcript 01534, with induction of expression both in *in vitro* and *in planta* appressorial samples (Table 3). The transcript 00302 is an ortholog of the *M. oryzae* P-type ATPase gene (*pde1*), required for the development of penetration hyphae and the proliferation of the fungus (Balhadère and Talbot, 2001) and was detected in the three *H. vastatrix* libraries at relatively constant expression levels. Orthologs of this gene were also identified in other rusts species (Broeker et al., 2006; Jakupović et al., 2006; Yin et al., 2009; Duplessis et al., 2011a), with elevated expression values in *P. pachyrhizi* appressoria (Stone et al., 2012). Among members of the Voltage-gated K^+^ Channel β subunit family are two transcripts (00184 and 00427) identified in the three *H. vastatrix* libraries, and one (04218) only identified in the H library but at higher Ra values.

### Metabolic pathways

The availability of nutrients for the fungus is very scarce at the early stages of the infection process and energy must be obtained from urediniospore contents. Carbohydrate metabolism by glycolysis/tricarboxylic acid cycle (TCA)/glyoxylate shuttle and lipids metabolism seems to be crucial to the success of the penetration process (Solomon et al., 2004). In the present study, orthologs of genes coding several key enzymes of glycolysis and TCA pathways were identified that presented higher Ra values in gU and Ap datasets (Supplementary Data 12, panels A and E). Polyols and trehalose are among the sugars mobilized during germination (D'Enfert et al., 1999; Voegele and Mendgen, 2003). One of the major roles of trehalose seems to be the regulation of glycolysis. In the trehalose biosynthetic pathway, the intermediate trehalose 6-phosphate plays an important metabolic regulatory role by controlling glycolysis through hexokinase. In *H. vastatrix*, transcript 00156, orthologous of a hexokinase, is upregulated in Ap according to RT-qPCR results. Two transcripts (04402 and 04553) were identified orthologous of trehalose-6-phosphate synthase genes in *M. larici-populina* (gene 33497) and *P. graminis* f. sp. *tritici* (PGT_06208), and RT-qPCR results showed the accumulation of transcript 04402 in appressoria. *H. vastatrix* transcript 00704, an ortholog of a neutral trehalase (*M. larici-populina* gene 116200), was detected in the three libraries, RT-qPCR showing a peak of expression in the appressoria and at 21 days after inoculation, suggesting a close control of trehalose/trehalose-6-P levels at these stages.

The glycolysis pathway leads to the production of pyruvate after convertion into acetyl-CoA. This pathway is fundamental for cell survival since it provides intermediate metabolites and other important small molecules, such as ATP and NADH. In the present dataset, all enzymes involved in this pathway were detected (Supplementary Data 12, panel A). A close connection between glycolysis and other pathways such as pentose phosphates and β-oxidation suggests the existence of a tight control of carbohydrate mobilization and utilization. Dihydroxy acetone phosphate, produced by aldolase by the glycerol-3-phosphate shuttle, can lead to the formation of glycerol (Supplementary Data 12, panel C) (Cronwright et al., 2002). In *H. vastatrix*, transcript 08812, ortholog of a glycerol 3-phosphate dehydrogenase, was identified in the Ap library. RT-qPCR analysis (Table 3) further revealed its expression during pre- and post-penetration events, strongly decreasing at late colonization stages. Similarly, transcript 01400 (glycerol 3-phosphatase gene ortholog), was accumulated in gU and Ap samples (Table 3). In *Saccharomyces cerevisiae*, the role of glycerol has been described in the maintenance of the cytosolic redox state (Cronwright et al., 2002). Besides, in fungi such as *Magnaporthe* or *Colletotrichum*, the important turgor pressure built in appressoria is mediated by the accumulation of very large amounts of glycerol in the cell (de Jong et al., 1997; Soanes et al., 2012). In *H. vastatrix*, transcripts with homology to glycerol 3-phosphatase and NAD^+^-dependent glycerol 3-phosphate dehydrogenase (transcripts 01400 and 06448, respectively) showed higher expression during germination and appressoria formation according both to 454 pyrosequencing and RT-qPCR results. Increased levels of these enzymes were also described in *P. pachyrhizi* at the appressorial stage (Stone et al., 2012). The glycerol formed is metabolized by the action of a glycerol kinase (transcripts 06788 and 09049) the expression of which is also observed during appressorial formation according both to 454 pyrosequencing and RT-qPCR results, suggesting the importance of the maintenance of glycerol contents. While the sum of Ra values suggests higher expression of genes related to metabolism in the H library, a higher number of genes was identified in the Ap library (Figure 2).

Beyond the glycerol-3-phosphate shuttle, glycerol generation may also be achieved by the mobilization of storage lipids through degradation of triacylglycerol by triacylglycerol lipases (EC 3.1.1.3) (Thines et al., 2000). In fact, flexibility in lipid metabolism and ability to divert intermediates from glycolysis identified in *M. oryzae* was suggested to be important for rapid glycerol accumulation during appressorium development (Dean et al., 2005). In this study, the results suggested a high rate of lipid metabolism during germination and appressoria formation. Among the 16 putative lipases (transcripts 00223, 00443, 00530, 00606, 01163, 01746, 01917, 02201, 04308, 06521, 06529, 07167, 07216, 07621, and 09011), 12 were found in the gU library and nine in the Ap library, while only two transcripts were expressed in the H library (Supplementary Data 4). Lipid metabolism is important for ATP generation and as a source of intermediates to secondary metabolic pathways. Fatty acids are oxidized by β-oxidation, a pathway that has been referred crucial for appressorium formation, in addition to the glyoxylate cycle, to enable utilization of acetyl-CoA for central carbohydrate metabolism (Kretschmer et al., 2012; Soanes et al., 2012). The present study enabled the identification of orthologs of all genes involved in β-oxidation pathways in *M. larici-populina* and *P. graminis* f. sp. *tritici* (Supplementary Data 12, panel D). The comparison among the three *H. vastatrix* libraries revealed that fatty acid degradation increased in Ap as indicated by the increased expression of transcripts coding for several β-oxidation enzymes such as long-chain fatty acid CoA ligase (transcript 00175), acyl-CoA dehydrogenase (transcript 01629), enoyl CoA hydratase (transcript 01055), 3-hydroxyacyl-CoA dehydrogenase (transcript 01628), and 3-ketoacyl-CoA thiolase (transcript 00191). A similar profile was detected for acyl-CoA oxidase (transcript 00622). RT-qPCR profiles for these transcripts further revealed a second peak of expression at 2 days for transcripts 00191 and 01628, and at 7 days for transcript 00175. Transcripts 01594, 01988 and 07400 are orthologs of *M. oryzae* carnitine acetyl-transferase gene (*crat1*), involved in transport of peroxisomal acetyl-CoA. *M. oryzae* deletion mutants for this gene show reduced appressoria melanisation, and are not able to elaborate penetration pegs or infection hyphae (Ramos-Pamplona and Naqvi, 2006). Interestingly, in *H. vastatrix* these transcripts were only identified in gU and Ap libraries, further suggesting their potential involvement in appressorium-mediated infection. RT-qPCR analyses illustrate different expression profiles for these three transcripts: while 07400 is induced during appressoria formation both *in vitro* and *in planta*, with a second peak of induction at 7 days, transcripts 01594 and 01988 are mostly over-expressed during hyphal colonization of host tissues, after 2 days for transcript 01594 and as early as appressoria differentiation for transcript 01988 (Table 3).

The glyoxylate cycle provides means for cells to assimilate two-carbon compounds into the TCA cycle and channel these via gluconeogenesis to the biosynthesis of glucose, (Supplementary Data 12, panel E). Generally, induction of the glyoxylate cycle indicates that a cell is using lipid metabolism as its predominant source for ATP generation, involving β-oxidation of fatty acids and the production of acetyl CoA. In *H. vastatrix*, results showed the presence of transcripts coding for all enzymes of the glyoxylate cycle. Ra values, as well as RT-qPCR analysis for transcripts 00491, 00717, 01133, 01266, and 08833, suggest an increasing level of expression during appressoria formation. The fact that glyoxylate cycle allows the connection between lipid and carbon metabolism may be particularly important for foliar pathogenic fungi that need to germinate and develop specific infection structures before having access to plant nutrients (Wang et al., 2003).

### Signaling

A total of 25 *H. vastatrix* transcripts presented homology to genes involved in signaling, whose mutants in various fungi exhibit either loss of pathogenicity or reduced virulence phenotype (recorded in the PHI database) (Supplementary Data 5). In the cAMP pathway, transcripts 01548 and 08827 are orthologs of pathogenicity-required adenylyl cyclase (*cdc35*) and adenylate cyclase (*cac1*) genes, respectively from *Candida albicans* (Rocha et al., 2001) and *Colletotrichum lagenarium* (Yamauchi et al., 2004), necessary for filamentous growth. Matching the relevance of this gene for spore germination and differentiation of infection structures from appressoria, RT-qPCR analysis showed the accumulation of transcript 08827 at the appressorial stage and of transcript 01548 at early infection stages, from urediniospore germination until 3 days (Table 3). Also, transcripts 00898 and 01431 are orthologs of the *Colletotrichum trifolii* pathogenicity-required catalytic subunit of cyclic AMP-dependent protein kinase gene (*pkac*), necessary for penetration and sporulation (Yang and Dickman, 1999). RT-qPCR profiles showed induction of their expression in appressorial samples both obtained *in vitro* and *in planta* (Table 3), compatible with the involvement of these genes in penetration. Another protein kinase involved in the cAMP pathway is the *M. oryzae*/*C. trifolii* pathogenicity-required *cpkA* gene, ortholog of *H. vastatrix* transcript 06436, required for appressorium formation and pathogenesis (Mitchell and Dean, 1995; Yang and Dickman, 1999). RT-qPCR results further suggested an activation of the expression of this transcript in the appressorial stage (Table 3).

Several MAP kinases and serine/threonine kinases were identified, and RT-qPCR results further corroborated induction of their expression in germinating urediniospores and/or in appressoria. By instance, orthologs of the *Ustilago maydis kpp6* and *ubc3* genes (Mayorga and Gold, 1999; Brachmann et al., 2003), the *Cryphonectria parasitica cpmk1* gene (Park et al., 2004), the *Claviceps purpurea cpmk1* gene (Mey et al., 2002) and the *Cryptococcus neoformans* var. *grubii hog1* gene (Bahn et al., 2005) were identified. *H. vastatrix* transcripts 06883 and 07140, orthologs respectively of the *C. purpurea* and of *C. parasitica cpmk1* genes, were both identified only in the gU library. RT-qPCR analysis showed distinct expression profiles, with transcript 06883 induced at pre-penetration stages only, and transcript 07140 expressed at all infection stages except late colonization and resting urediniospores (Table 3). On the contrary, both the *ubc3* type transcript 00373 and the *hog1* type transcript 00616 were identified in the three libraries showing stable expression profiles, as corroborated by RT-qPCR results (Table 3). In *M. oryzae*, the Pmk1 MAP-kinase pathway has a major role in controlling appressorium morphogenesis (Soanes et al., 2012). Also, two MAPK kinases (MAP2K) were identified, corresponding to transcripts 01932 and 01813, of the Ste7 and the Mkk1 types respectively (Hamel et al., 2012), both of them identified in the gU and Ap libraries. RT-qPCR results further showed that the expression of transcript 01932 is observed at early infection stages (Table 3). The *H. vastatrix* transcripts 00125 and 00489, and 00409, orthologs respectively of the of the *M. oryzae* MAP kinase-regulated *gas1* and *gas2* genes (Xue et al., 2002), were identified in the three libraries, with high expression levels (Ra > 1) in the gU and Ap libraries. RT-qPCR results corroborate the induction of expression of these transcripts in germinating urediniospores and in appressoria, with a second peak of expression recorded for transcripts 00409 and 00489 respectively at 2 and 3 days (Table 3). Interestingly, transcript accumulation for *gas* orthologs in *P. pachyrhizi* and *Uromyces appendiculatus* purified haustoria were also reported (Link et al., 2013). The present results indicated that *gas* expression is not solely related to the rust haustorial infection structure, but also to earlier stages such as spore germination. A group of *H. vastatrix* transcripts show homology to G protein subunits genes from the PHI base. Heterotrimeric G-proteins transduce extracellular signals to various downstream effectors (e.g., MAP kinases) in eukaryotic cells. Transcript 06565 shows homology to the *Cryptococcus neoformans* virulence-related *gpa1* gene (Alspaugh et al., 1997) and to the *M. larici-populina* heterotrimeric G-protein α subunit 3 gene (*gpa3*) (Duplessis et al., 2011a). In *H. vastatrix*, transcript 06565 was only detected in the gU library, suggesting an involvement in pre-penetration events, in agreement with the profile of its *M. larici-populina* ortholog (gene 47478) (Duplessis et al., 2012). Orthologs of G-protein β subunit genes involved in appressorium formation, including genes *mgb1* from *M. oryzae* (Nishimura et al., 2003), *cgb1* from *Cochliobolus heterostrophus* (Ganem et al., 2004) and *Bpp1* from *U. maydis* (Müller et al., 2004), were identified in *H. vastatrix* (transcripts 00968 and 02022 for *mgb1* and 07174 for *cgb1*/*Bpp1*), all of them in the gU library, indicative of a possible role in appressoria formation in *H. vastatrix*. RT-qPCR expression profiling further showed induction of transcripts 02022 and 07174 during early pre-penetration events (Table 3).

### Other genes identified in rust transcriptomic/genomic studies

Several orthologs of *U. fabae in planta*-induced genes (PIGs) were identified in *H. vastatrix* (Supplementary Data 13). The *U. fabae* PIGs genes showed induced expression in *Vicia faba* infected leaves as compared to germinating urediniospores (Jakupović et al., 2006). The majority of *U. fabae* transcripts with a predicted function have orthologs in *H. vastatrix* (Supplementary Data 13).

A comparison of *H. vastatrix* genes to *P. pachyrhizi* genes expressed in germinating urediniospores (Posada-Buitrago and Frederick, 2005) reveals that the two most expressed *P. pachyrhizi* genes, *Pp0104* and *Pp0417*, have no significant homologies in *H. vastatrix* (Supplementary Data 14). In the same way, the comparison of the 9234 *H. vastatrix* transcript to a collection of ESTs and proteins differentially expressed in *P. pachyrhizi* appressoria (Stone et al., 2012) shows a very limited number of genes in common between both studies (Supplementary Data 14). Interestingly however, the comparison of 4492 *P. pachyrhizi* haustorial ESTs (Link et al., 2013) to the 9234 *H. vastatrix* transcripts identified 1668 hits to 1132 unique *H. vastatrix* transcripts. Half of them corresponded to *H. vastatrix* transcripts not detected in the H library (Supplementary Data 14). A similar situation was observed when comparing the 7561 *U. appendiculatus* haustorial ESTs (Link et al., 2013) to the *H. vastatrix* transcripts (data not shown).

Among the 156 *M. larici-populina* annotated genes that are >10-fold up-regulated in infected leaves as compared to urediniospores (Duplessis et al., 2011a), only 22% have orthologs in the present *H. vastatrix* dataset, including 12 transporters (mostly sugar and ion transporters), 10 secreted proteins and six glycoside hydrolases (Supplementary Data 4, columns Y-AA). Among the 235 *P. graminis* f. sp. *tritici* annotated genes that are the >10-fold up-regulated in infected leaves as compared to urediniospores (Duplessis et al., 2011a), 49% have orthologs in the present *H. vastatrix* dataset, although half of these are predicted ribosomal genes. Unlike for *M. larici-populina*, none of these *P. graminis* f. sp. *tritici* genes include glycoside hydrolases or secreted proteins and only two transporters were identified.

An expanded number of multigene families have been reported in *M. larici-populina* and *P. graminis* f. sp. *tritici* as compared to other Basidiomycetes (Duplessis et al., 2011a). Among those expanded families, the number of *H. vastatrix* transcripts-based predicted genes is higher than those for *M. larici-populina* or *P. graminis* f. sp. *tritici* for the major facilitator superfamily, helicase or chitinase, and under-represented for families such as serine/threonine protein kinase and sugar transporter (Table 6). While the current study does not cover all differentiation stages of the *H. vastatrix* life cycle and transcripts expressed at low level may not be represented, it is interesting to note that some gene families are over-represented in comparison to annotated genome sequences.

**Table 6:**
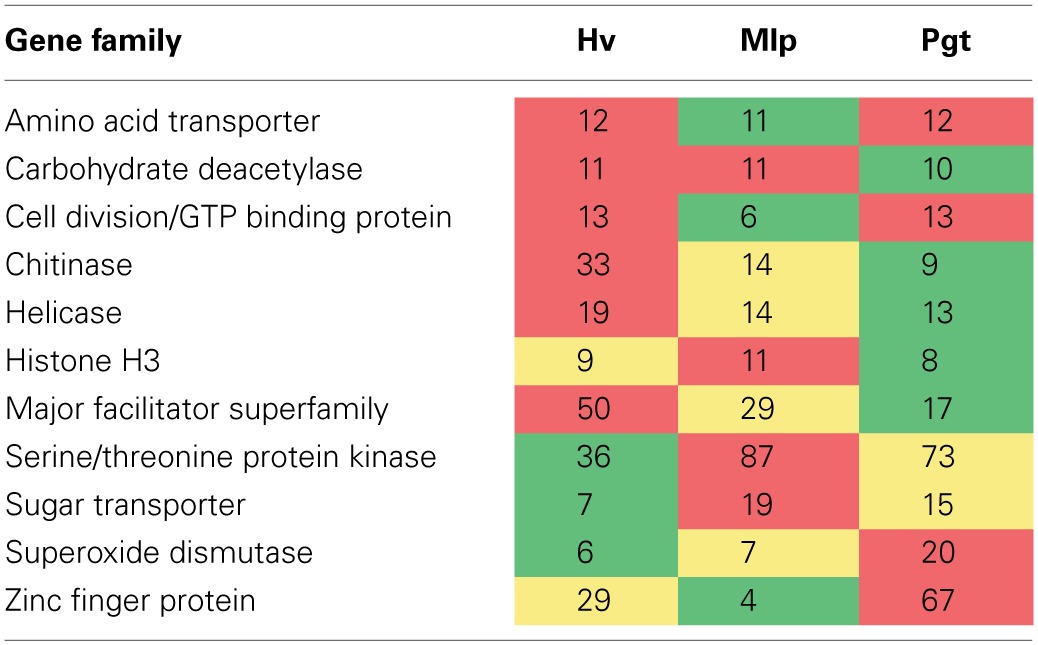
**Comparison of the number of *Hemileia vastatrix* (Hv) genes to the number of members of gene families in *Melampsora larici-populina* (Mlp) and *Puccinia graminis* f. sp. *tritici* (Pgt) reported (Duplessis et al., 2011a) as expanded in relation to other Basidiomycetes**.

*Colour scale: green to red denote lowest to highest number of genes for each gene family*.

## Conclusions

In this study, 7894 contigs were obtained by 454 pyrosequencing of cDNA from *H. vastatrix* germinating urediniospores and appressoria. These transcripts, along with 4465 *in planta* expressed contigs (Fernandez et al., 2012), were assembled into 9234 annotated transcripts. This number represents an important fraction (>50%) of the genes predicted in rust sequenced genomes so far (Duplessis et al., 2012). In addition, this elevated gene number for *H. vastatrix* is corroborated by other database comparisons, such as the core fungal genes database (FUNYBASE), the carbohydrate-active enzymes (CAZy) database or the Transporter Classification Database (TCDB). Database comparisons further indicate that half of these transcripts (4707) present no significant homology to genomic or transcriptomic data from other rusts, potentially representing novel or very divergent *H. vastatrix* genes.

Annotation of *H. vastatrix* transcripts and comparison of their relative abundance in each of the three sampling stages suggest a particularly active metabolism, translational activity, production of new structures and signaling in appressoria and intense transport, secretory activity and cellular multiplication in germinating urediniospores (Figure 2). Transcripts encoding putative carbohydrate-active enzymes and different types of transporters are more expressed in germinating urediniospores and appressoria, and lesser at late infection stages. Among transcripts involved in metabolic pathways, an active lipid metabolism was observed at pre-penetration stages compared to late infection stages, while aminoacid and carbohydrate metabolism was more active in post-penetration samples. Moreover, the homology of *H. vastatrix* transcripts to genes known to be involved and/or required for pathogenicity in other fungal plant pathogens, namely in appressoria-mediated infection, enabled the identification of an array of putative pathogenicity factors, a large proportion of which are expressed as early as during germ-tube elongation. Also, while melanized cuticle-breaching appressoria have been thoroughly investigated over the last few decades, namely in *M. oryzae* and *Colletotrichum* spp. (Deising et al., 2000; Kleemann et al., 2012), the present study represents an important insight into genes expressed in non-melanized stomata-penetrating appressoria. To this end, induction of expression of genes related to the production of carbohydrate-active enzymes and to the accumulation of glycerol in germinating urediniospores and appressoria suggests that combined lytic and physical mechanisms are involved in appressoria-mediated penetration of coffee leaf stomata.

This early activation of signaling, transport and secretory pathways suggests a precocious plant-fungus dialogue, which is corroborated by the possible induction of an hypersensitive reaction in stomatal cells of some resistant coffee varieties as early as at the appressorial stage (Silva et al., 2002; Ganesh et al., 2006; Diniz et al., 2012), thus prompting further studies targeting the identification of virulence/avirulence factors (and their resistance/susceptibility conterparts) expressed at these early stages of the plant-fungus interaction.

## Author contributions

This study was conceived and directed by Pedro Talhinhas, Helena G. Azinheira, Sébastien Duplessis, Maria do Céu Silva, and Diana Fernandez. The laboratorial experiments were conducted by Pedro Talhinhas, Helena G. Azinheira, Andreia Loureiro, Sílvia Tavares, and Anne-Sophie Petitot. 454-pyrosequencing was conducted by Julie Poulain and Corinne Da Silva. Bioinformatic analyses were conducted by Bruno Vieira, Emmanuelle Morin, and Octávio S. Paulo. Biological interpretation of bioinformatics analyses were conducted by Pedro Talhinhas, Helena G. Azinheira, Andreia Loureiro, Sílvia Tavares, Sébastien Duplessis, and Diana Fernandez. Pedro Talhinhas, Helena G. Azinheira, Andreia Loureiro, Sílvia Tavares, Dora Batista, Octávio S. Paulo, Sébastien Duplessis, Maria do Céu Silva, and Diana Fernandez wrote the paper. All authors read and approved the final manuscript.

### Conflict of interest statement

The authors declare that the research was conducted in the absence of any commercial or financial relationships that could be construed as a potential conflict of interest.
