# Supplementary figures and images for: Overview of the functional virulent genome of the coffee leaf rust pathogen Hemileia vastatrix with an emphasis on early stages of infection
Source: Front Plant Sci. 2014 Mar 14;5:88. doi: 10.3389/fpls.2014.00088 (PMC3953675; doi:10.3389/fpls.2014.00088)

#### D. $\beta$ -oxidation

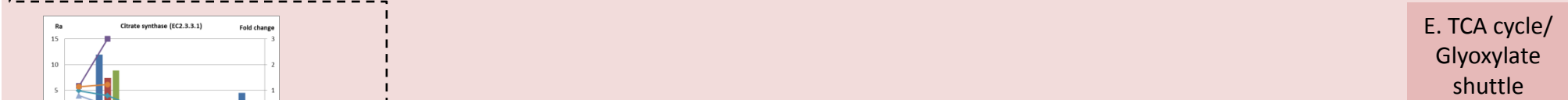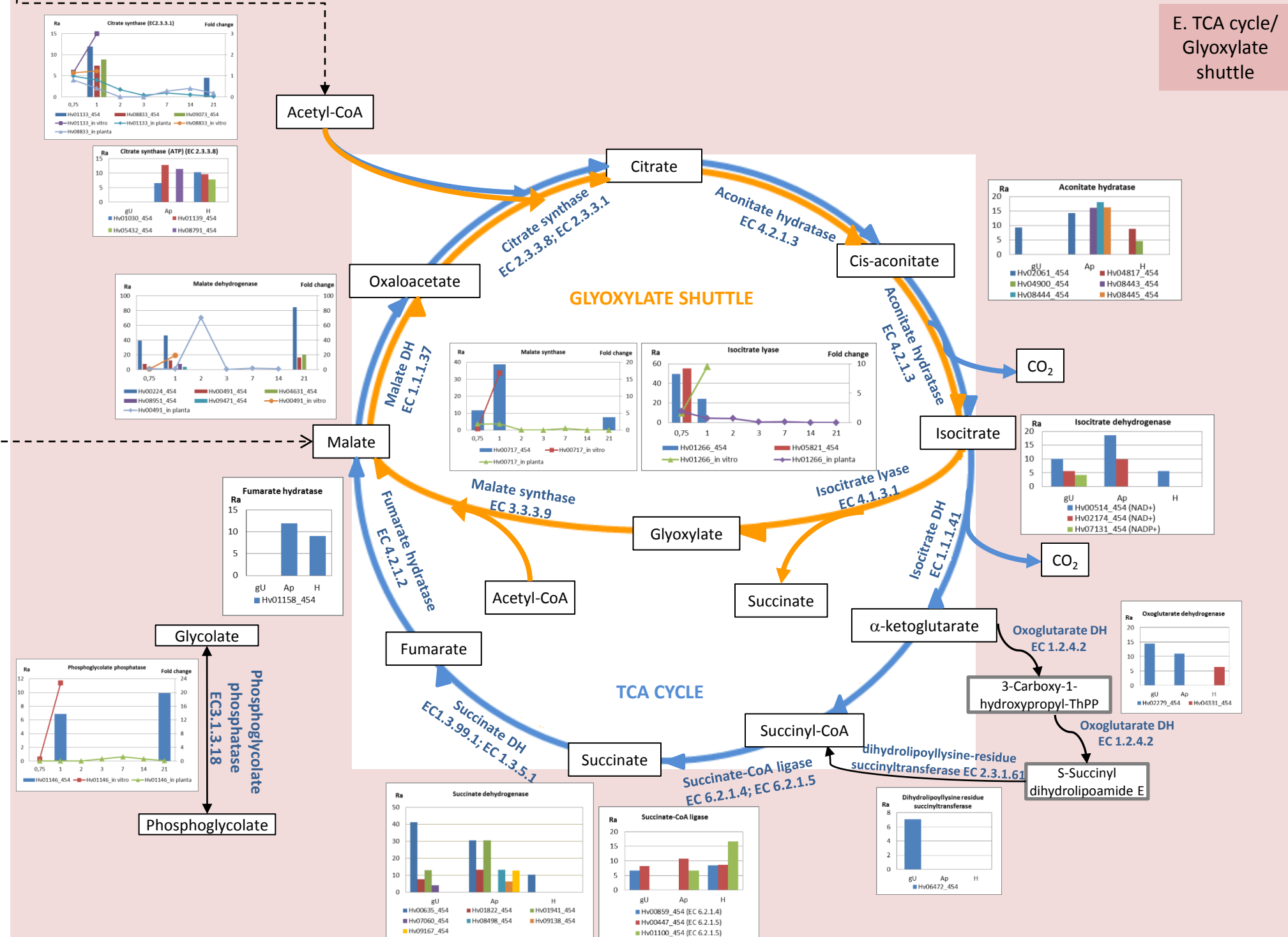

Supplement: Supplementary file 1 [file Presentation1.ZIP › 81604_Azinheira_Data_Sheet_12.PDF]
